# Rapid clinical improvement of atopic dermatitis in an Omalizumab treated patient

**DOI:** 10.1186/s12948-019-0109-z

**Published:** 2019-03-12

**Authors:** Susanna Bormioli, Andrea Matucci, Laura Dies, Francesca Nencini, Francesca Grosso, Enrico Maggi, Alessandra Vultaggio

**Affiliations:** 1 Immunology and Cellular Therapy, Azienda Ospedaliero-Universitaria Careggi, Largo Brambilla 3, 50134 Florence, Italy; 2 Immunoallergology Unit, Department of Biomedicine, Azienda Ospedaliero-Universitaria Careggi, Florence, Italy

**Keywords:** Omalizumab, Atopic dermatitis, Anti-IgE, Quality of life

## Abstract

**Background:**

Atopic dermatitis is a chronic inflammatory skin disorder, whose symptoms and severity grossly depend on individual trigger factors. The majority of patients are satisfactorily treated with emollients together with topical and systemic therapies. However, treatment failure or long-term side effects with conventional treatment options can be a significant clinical problem. Recently, novel therapeutic approaches focus on targeting skewed immune responses providing a more effective, and less harmful approach. Among them, variable success has been reported using Omalizumab, when used in combination with classic therapies. This report describes an interesting case of severe adult onset difficult-to-treat atopic dermatitis dramatically improved in response to treatment with Omalizumab.

**Case presentation:**

We present a case of an adult male with severe allergic atopic dermatitis, with concomitant involvement of the face, neckline, trunk and forearms and systemic symptoms such as diarrhoea with important decrease of his daily quality of life. The patient had been prescribed oral steroids in addition to anti-histamines to no avail. Due to lack of response to classic therapies, strict diet, as well as to treatment with intravenous corticosteroids, an off-label treatment with Omalizumab based on patient weight and total IgE value was proposed. Clear clinical results were observed after only a few weeks with regards to systemic symptoms, and just after 2 months of treatment in regards to skin involvement.

**Conclusions:**

In the majority of treated patients the clinical improvement of cutaneous manifestations is expected after several months of treatment, as skin manifestations are the consequence of a chronic inflammatory process. The outstanding rapid response observed in this case as well as the persistence of the clinical remission suggests that the block of the IgE pathways modulate functions of cells involved in the pathogenic mechanisms of chronic skin inflammation but also in the acute phases observed in the flare-ups of the disease.

## Background

Atopic dermatitis (AD) is a multifactorial cutaneous inflammatory disease, manifesting with a chronic relapsing trend. It is characterized by eczematous lesions, skin xerosis, and intense pruritus that can present in a mild, moderate, or severe form. Prevalence in adults is 7–10% and can arise at any age both as a disease flare-up but also as “very late onset” AD. Severe forms of AD have an important impact on patients’ quality of life (QoL), and often require long-term immunosuppressive drugs. The complex interplay among barrier deficiency and immunological mechanisms contributes to the development, progression and chronicity of the disease. From a pathogenic point of view, T helper (Th)2 cells play a fundamental role through the production of interleukin (IL)-4, IL-5, IL-13 by influencing the recruitment of eosinophils and inducing the production of IgE antibodies [1, 2]. Numerous clinical trials of biological agents are currently being conducted in AD patients. Omalizumab, a humanized IgG1k monoclonal antibody that binds to free IgE, is now accepted for the treatment of severe asthma and chronic spontaneous urticaria, and used, in a off-label strategy, in resistant severe AD [3]. The mechanisms of action of Omalizumab are not completely defined but certainly related not only to the inhibition of mast cell/basophils activation but also to the modulation of several cells such as dendritic cells (DC), T and B lymphocytes, involved in the pathogenesis of chronic allergic inflammation [4, 5].

## Case presentation

**Fig. 1:**
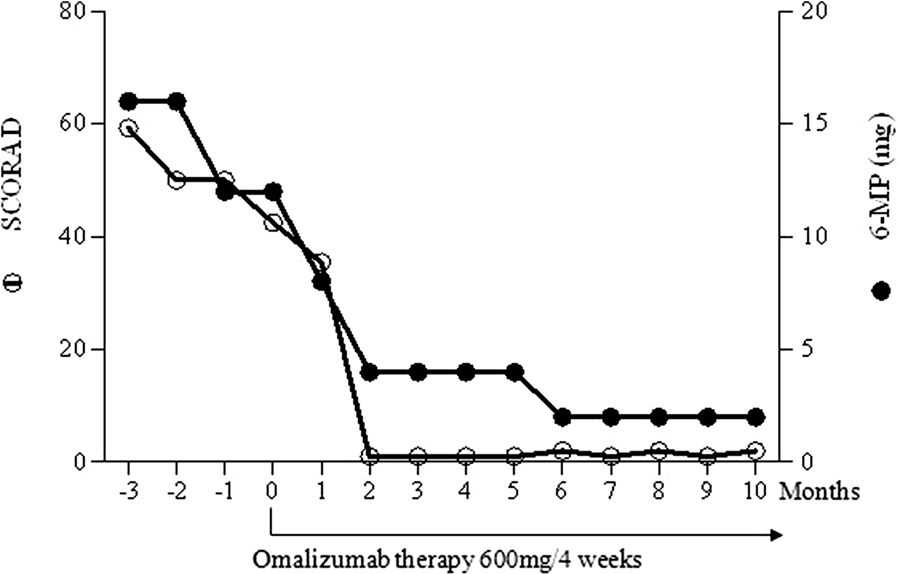
Rapid SCORAD improvement and corticosteroid tapering in regards to Omalizumab therapy

**Fig. 2:**
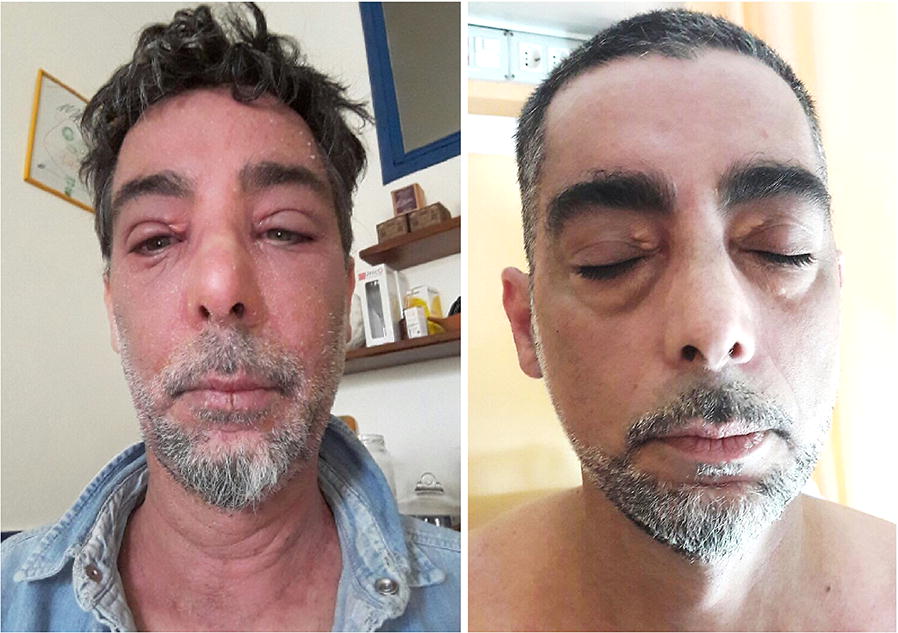
Patient at T0 and after two months of Omalizumab treatment

We present a case of a 53-year-old male, a professional chef, with a personal history for allergic rhino-conjunctivitis. He began showing initial signs of AD in 2015, with involvement of the face, neckline, trunk and forearms. The allergological work-up was positive for pollens, foods, latex. Due to a severe exacerbation of dermatitis, the patient was referred to our attention on January 2017. The patient had been prescribed oral steroids at an average daily dose of 8–12 mg of 6-methyl prednisolone, raised during flare-ups, in addition to anti-histamines (cetirizine 10 mg/day), to no avail. At this time he presented with a sub-erythrodermic state and a SCORing Atopic Dermatitis (SCORAD) of 59.3. In vitro testing (ImmunoCAP Thermofisher, Upssala, Sweden) found IgE serum levels of 433 kU/L, and specific IgEs positive for pollens [Grass pollens (21 kUA/L); Olive tree (6.1 kUA/L); Cypress tree (4.4 kUA/L); Parietaria officinalis (6.04 kUA/L)]; Dermatophagoides pteronyssinus (27.8 kUA/L); and foods [hazelnuts (26.3 kUA/L); peanuts (18.4 kUA/L); wheat (6.4 kUA/L); tomato (31.4 kUA/L); onion (53.3 kUA/L); apple (51.2 kUA/L)]. In vitro testing for molecular allergens was positive for Prup3 (72.7 kUA/L) and Betv2 (8.6 kUA/L). Peripheral blood eosinophil count was 2.8% (260 cells/mmc). Daily episodes of diarrhoea was referred despite the diet restrictions. Frequent severe exacerbations occurred due to the exposure to cooking vapour during the work activity. No history of anaphylactic reactions was referred. Given the absence of previous acute hypersensitivity reactions to foods, but taking into account the high levels of Prup3, and specific food IgEs, the patient followed a pseudo-allergen free diet and exclusion of grains and tomatoes for 6 months. To induce a rapid improvement of symptoms, three consecutive days of treatment with 80 mg of 6-methyl prednisolone i.v. were administered, progressively tapered over the course of 3 weeks to a minimal daily dose of 8 mg. However, a rapid relapse of the skin legsions, and severe pruritus was observed. Considering the lack of response, treatment with Cyclosporine A (CoA) 3 mg/kg body weight (kg 75) was started. The patient was advised to implement strict environmental, hygiene, and diet restrictions based on the test results. After 3 months the patient found no significant improvement, with a SCORAD of 42.49. Due to severe clinical presentation, the weak response to conventional therapy and CoA, an off-label treatment with Omalizumab 600 mg every 4 weeks based on patient weight and total IgE value was proposed, after obtaining written informed consent from the patient and the local Ethical Committee approval. Unexpectedly, clinical results were observed after just about 2 months of treatment as confirmed by a SCORAD of 1, and a clear improvement of QoL (Fig. 1). Also, gastrointestinal symptoms disappeared after 3 weeks from the beginning of treatment. Steroid dose was progressively tapered to 2 mg of 6-methyl prednisolone in anticipation of complete suspension, and a gradual reduction of CoA until a stable maintenance dose of 1 mg/kg/day was reached. The patient has since returned to work as a chef without complications, and has begun eating grains again with no gastro-intestinal problems nor relapse of dermatitis, but only occasional mild pruritus (just one occurrence). After 1 year of Omalizumab treatment, the patient maintains a good control for both skin and gastrointestinal symptoms (Fig. 2).

## Discussion and conclusions

Despite the case represents an example of Omalizumab’s efficacy in AD, in the majority of treated patients the clinical improvement of cutaneous manifestations is expected after several months of treatment. This is in agreement with the fact that skin manifestations are the consequence of a chronic inflammatory process in which several immune and structural cells as well as their products are involved [1]. The rapid response observed in this case as well as the persistence of the clinical remission suggests that the block of the IgE pathways modulate functions of cells involved in the pathogenic mechanisms of chronic skin inflammation but also in the acute phases observed in the flare-ups of the disease. The capacity of Omalizumab to block allergen-specific IgEs enables the explanation of the relatively fast clinical response in which mast cell activation is certainly involved. Some doubts can be raised taking into account that membrane bound IgE linked to FcεRI persist for several months and are not recognized by Omalizumab [6]. However, as recently demonstrated, Omalizumab is also able to induce a detachment of IgE fixed to FcεRI on the membrane of cells such as DC, by reducing their capacity to activate T cells [7]. The final result being a down regulation of the chronic inflammatory process, characteristic of AD. The complex mechanisms of Omalizumab’s action may explain the rapid and long lasting clinical effects and an exponential improvement of the QoL observed in our patient.

Currently, the use of Omalizumab in patients with AD is still under discussion, and more studies are needed to better define the efficacy of this drug and its target population.

In conclusion, we would like to include the following take home points:

1. A significant percentage of patients suffering from atopic dermatitis are unresponsive to conventional therapy.
2. Omalizumab is effective in severe atopic dermatitis non responsive to conventional treatment.
3. At least in a subgroup of patients, the clinical improvement of dermatitis after treatment with Omalizumab occurs very early.
4. The clinical response to Omalizumab persists over the course of treatment.
5. Omalizumab treatment allows to reduce the steroid and immunosuppressor doses.
6. Omalizumab is able to significantly improve the atopic dermatitis patient’s Quality of Life.

## Abbreviations

AD: atopic dermatitis
QoL: quality of life
Th: T Helper cell
IL: interleukin
DC: dendritic cell
SCORAD: SCORing Atopic Dermatitis
CoA: cyclosporine A
